# Supplementary material for: Angiotensin Converting Enzyme Inhibitors May Increase While Active Vitamin D May Decrease the Risk of Severe Pneumonia in SARS-CoV-2 Infected Patients with Chronic Kidney Disease on Maintenance Hemodialysis
Source: Viruses. 2022 Feb 22;14(3):451. doi: 10.3390/v14030451 (PMC8951398; doi:10.3390/v14030451)
Supplement: Supplementary file 1 [file viruses-14-00451-s001.zip › viruses-1568936-supplementary.pdf]

**Table S1** Treatment and clinical course during hospitalization

| Variable | ALL | MILD CHANGES | SEVERE CHANGES | *p value* * |
|---|---|---|---|---|
| N | 85 | 47 | 38 | |
| **Treatment** n (%) | | | | |
| Remdesivir | 5 (5.88) | 1 (2.13) | 4 (10.53) | 0.10 |
| Convalescent plasma | 17 (20.0) | 5 (10.64) | 12 (31.58) | 0.02 |
| Corticosteroids | 47 (55.29) | 20 (42.55) | 27 (71.05) | 0.008 |
| Oxygen therapy | 60 (70.59) | 24 (51.06) | 36 (94.74) | <0.001 |
| < 15 l/min | 48 (57.14) | 21 (44.68) | 27 (72.97) | 0.01 |
| >15 l /min | 12 (14.29) | 3 (6.38) | 9 (24.32) | 0.02 |
| Antibiotic treatment | 62 (73.81) | 29 (63.04) | 33 (86.84) | 0.004 |
| Red blood cells transfusion | 16 (18.82) | 8 (17.02) | 8 (21.05) | 0.59 |
| LMWH | 76 (89.41) | 40 (85.11) | 36 (94.74) | 0.15 |
| **Outcome** | | | | |
| Death in hospital n (%) | 23 (27.06) | 10 (21.28) | 13 (34.21) | 0.18 |
| Day of death - median (IQR) | 10 (3-16) | 15 (10-20) | 4 (3-10) | 0.02 |
| Days of hospitalization ** - mean (SD) | 17.77 (7.37) | 15.92 (5.76) | 20.52 (8.67) | 0.01 |

Legend: LMWH, low molecular weight heparin. * - mild changes group vs. severe changes group. ** - survivals
